# The Sense of Commitment in Human–Robot Interaction

**DOI:** 10.1007/s12369-016-0376-5

**Published:** 2016-09-20

**Authors:** John Michael, Alessandro Salice

**Affiliations:** 1 Department of Cognitive Science, Central European University, Oktober 6 Utca 7, Budapest, 1051 Hungary; 2 Center for Subjectivity Research Copenhagen University, Njalsgade 140-142, Building 25, 5th floor, 2300 Copenhagen, Denmark

**Keywords:** Commitment, Trust, Human–robot interaction, Joint action, Cooperation, Coordination

## Abstract

The sense of commitment is a fundamental building block of human social life. By generating and/or stabilizing expectations about contributions that individual agents will make to the goals of other agents or to shared goals, a sense of commitment can facilitate the planning and coordination of actions involving multiple agents. Moreover, it can also increase individual agents’ motivation to contribute to other agents’ goals or to shared goals, as well as their willingness to rely on other agents’ contributions. In this paper, we provide a starting point for designing robots that exhibit and/or elicit a sense of commitment. We identify several challenges that such a project would likely confront, and consider possibilities for meeting these challenges.

## Introduction

There is a vast potential for robots to assist humans in joint actions in many different domains, from disaster relief to health care, education, and manufacturing. As roboticists move forward in optimizing human–robot interactions in order to tap this potential, it may be fruitful to consider one particular question pertaining to the challenge of designing robots with whom humans can interact comfortably and productively in various kinds of joint action. Specifically: Is it possible to design robots that elicit and/or exhibit a sense of commitment—i.e. such that (i) humans agents are motivated by a sense of commitment toward them, (ii), human agents expect them to be motivated by a sense of commitment toward human agents, (iii) they are motivated by a sense of commitment toward human agents, and/or (iv) they expect human agents to be motivated by a sense of commitment toward them. To the extent that it is feasible to implement one or more of these forms of a sense of commitment in human–robot interaction, this could be useful insofar as it could enhance human agents’ willingness to rely on robots (e.g. to perform actions which depend upon a contribution from a robot and otherwise entail a risk), could motivate human agents to perform important actions which they otherwise might not perform (e.g. to practice new skills which they are learning with a robot collaborator, to take their pills regularly because they have made a commitment to their robot nurse that they will do so, or more generally, to promote therapeutic interactions—especially with regard to elderly people, cf. [1]).

In a nutshell, a sense of commitment could be highly useful in optimizing *human–robot* interactions, since the sense of commitment, as we will be arguing in a moment, serves important functions which are of great benefit in *human–human* interactions. In the following, we will address this issue in three steps: we will first (Sect. 2) lay out some conceptual preliminaries pertaining to commitment and the sense of commitment, to the social functions thereof, and to the ways in which commitment and the sense of commitment can be generated and sustained in human–human interactions. We will then (Sect. 3) identify and discuss a general conceptual challenge that arises when we examine the possibility of implementing commitment in the strict sense in human–robot interaction. We endorse a pragmatic approach to this challenge, which remains neutral as to whether commitment in the strict sense is possible in human–robot interaction, but which articulates the notion of a sense of commitment in such a way that it is clearly applicable *in principle* to human–robot interaction. Finally, (Sect. 4), we will identify several concrete factors which may be useful *in practice* for implementing the sense of commitment in human–robot interaction.

## Conceptual Preliminaries

### Commitment and the Sense of Commitment

When human agents are performing a joint action together, they typically have a sense of being committed to remaining engaged until the shared goal is achieved or until all participants have agreed to interrupt the joint action. Thus, to borrow Margaret Gilbert’s famous example, if two people are taking a walk together, they are likely to take themselves to be obligated to walk at a pace that the other can keep up with, to refrain from suddenly veering off into the woods without giving any explanation, and to wait for the other if she has to tie her shoe (cf. [2]). Correspondingly, each of them will typically expect that the other will also fulfill these obligations, and otherwise take herself to be entitled to reprimand her. In the canonical case, this is because each has given the other an assurance that they will remain committed (e.g. they have explicitly agreed to take a walk together). But even in the absence of explicit assurances, a more or less implicit sense of commitment can often be generated through bodily cues, subtle nuances of verbal communication or situational affordances, and may have very similar effects, and be experienced in very similar ways. We therefore find it useful to distinguish sharply between commitment, on the one hand, and the sense of commitment on the other. We will first offer a few remarks about commitment, and then turn our attention to the sense of commitment.

Despite its prevalence in everyday interactions, the phenomenon of commitment is surprisingly difficult to characterize. According to a standard philosophical conception, a commitment is a triadic relation among two agents and an action, where one of the agents is obligated to perform the action as a result of having given an assurance to the other agent that she would do so, and of the other agent’s having acknowledged that assurance under conditions of common knowledge [3–5]. We will refer to commitment in this standard philosophical sense as ‘commitment in the strict sense’. For example, Susie has an obligation to Jennifer to pick up the kids from school because she (Susie) has expressed her willingness to do so, and Jennifer has acknowledged this. In the canonical case, the expression is effectuated by means of the speech act of promising. As [4] puts it: ‘the utterance... predicates some future act A of the speaker S... [and] counts as the undertaking of an obligation to do A’ [4, p. 63].^1^

While this conception provides a clear characterization of commitments arising through verbal agreements, Michael et al. [6] have recently argued that it is not well-suited to explaining how and when an implicit sense of commitment can arise. To see why, consider the following example, adapted from one discussed by the philosopher Margaret Gilbert ([7], p. 9): Two factory workers, Polly and Pam, are in the habit of smoking a cigarette and talking together on the balcony during their afternoon coffee break. The sequence is broken when one day Pam waits for Polly but she doesn’t turn up. In this case, there has been no explicit agreement to smoke a cigarette and talk together every day, and yet one might nevertheless have the sense that an implicit commitment is in place, and that Polly has violated that implicit commitment. This will depend on further details about the case. For example, if Polly and Pam have smoked and talked together every day for two or three weeks, Polly might feel only slightly obligated to offer an explanation, but she would likely feel more strongly obligated if the pattern had been repeated for two or three years. Thus, it seems that mere repetition can give rise to an implicit sense of commitment. Similarly, one agent’s reliance on a second agent may give rise to a sense of commitment on the part of the second agent. If, for example, Polly and Pam always use Polly’s lighter, and Pam at some point even stopped bringing her own lighter, then Polly’s absence will completely undermine Pam’s goal of enjoying a pleasant cigarette break. In such a case, both parties are likely to think that an explanation, and perhaps even an apology, is all the more in order. Thirdly, one agent’s investment of effort or resources in a joint action may also give rise to a sense of commitment on the part of a second agent. If Pam, for example, must walk up five flights of stairs to reach the balcony where she and Polly habitually smoke together, Polly’s sense of commitment may be greater than if Pam only had to walk down the hall.

This example reveals that there are many situational factors which can give rise to a sense of commitment, and which can modulate the degree to which people feel or act committed, or expect each other to feel or act committed. The standard philosophical characterization of commitment in the strict sense does not provide any basis for identifying these factors. To fill this gap, Michael et al. [6] have recently proposed a framework based upon an analysis of the minimal structure of situations in which a sense of commitment can arise. This minimal structure can be expressed as follows:

- There is an outcome which an agent (ME) either desires to come about, or which is the goal of an action which ME is currently performing or intends to perform. We will refer to this outcome as ‘G’ (for ‘goal’).
- The external contribution (X) of a second agent (YOU) is crucial^2^ to bringing about G.

Clearly, conditions (a) and (b) specify a broader category than that of commitment in the strict sense. Nevertheless, situations with this structure may elicit a sense of commitment on the part of one or both agents. We propose to conceptualize the sense of commitment as follows:

ME has a sense that YOU is committed to performing X to the extent that ME expects X to occur because (a) and (b) obtain.

YOU has a sense of being committed to performing X to the extent that YOU is motivated by her belief that ME expects her to contribute X.

While this minimal structure is specified such that only one agent (ME) desires G and/or has G as a goal, there are many cases in which both agents desire G and/or have the goal G. In those cases, the sense of commitment may be mutual, with each agent having a sense of being committed as well as a sense that the other agent is committed.

It is also worth emphasizing that the two agents (ME and YOU) may differ with respect to their sense of commitment. Thus, ME may have a sense that YOU is committed even though YOU does not have a sense of being committed. Or YOU may have a sense of being committed even though ME does not have a sense that YOU is committed.

One virtue of the minimal approach is that it illuminates the conditions under which human agents feel and/or act committed, or feel and/or act as though a commitment were owed to them, even though they would not explicitly judge that a commitment is in place—i.e. it occurs when a sense of commitment is elicited. Moreover, the minimal approach makes it possible to identify various factors that modulate the degree of the sense of commitment, i.e. the extent to which agents are prepared to rely upon expectations about external contributions to their goals (and to shared goals), and the extent to which they are motivated to make contributions to others’ goals and to shared goals. Specifically, the minimal approach entails that any factor will raise the sense of commitment if it either raises the likelihood that ME will expect X to occur at least in part because she has the goal G or desires that G come about, or if it raises the likelihood that YOU will have a motivation to do X at least in part because YOU believes that ME expects this.

### Functions of Commitment and the Sense of Commitment

One important consequence of commitments, if they are credible, is that they make agents’ behavior *predictable* in the face of fluctuations in their desires and interests [8]. Thus, they make it possible to have expectations that one would not otherwise have about contributions that other agents are likely to make to one’s goals, or to have more reliable expectations than one would otherwise have. This is clearly a very useful social function, in part because it makes people willing to perform actions that they otherwise would not perform—to work, for example, given that somebody has made a commitment to pay them for it, or to lift one end of a heavy table that cannot be moved by one person alone. More specifically, having reliable expectations about others’ actions facilitates cooperation and coordination. In cooperation problems, such as the prisoners’ dilemma game [9], individuals are tempted to defect in order to maximize their own benefits but by cooperating maximize the overall group benefit. In coordination problems, on the other hand, such as the stag hunt game [10], two agents each maximize their individual benefits if they coordinate their actions, but get no benefit if they try to coordinate but fail to do so, and are therefore tempted to opt for a smaller benefit which does not depend upon coordinating with the other agent. Moreover, having reliable expectations about others’ contributions to one’s goals, or to shared goals, is also useful insofar as it may facilitate the planning of joint actions with complementary subplans, which depend upon and build upon each other, as well as the online coordination of joint actions among multiple agents [11, 12].

However, making credible commitments is not a trivial matter. When one makes a commitment to perform a particular action, one forecloses the possibility of performing other actions (or no action at all). But if some unforeseen alternative option arises which maximizes one’s interests and is thus more desirable than the action to which one is committed, why should one not revise one’s plan and take the alternative option? In other words, what motivates one to honor a commitment to perform an action that is not in one’s best interest? Indeed, this issue is even more serious than it appears at first glance, insofar as the flipside of motivation is credibility: why should one agent expect some other agent to remain committed to a particular action if that second agent’s desires or interests change? And if that second agent cannot be expected to honor her commitment, then it would be unwise to rely on it. Hence, in the absence of a motivation to honor a commitment, it is unclear how that commitment could perform the function of generating and/or stabilizing expectations about contributions agent will make to the goals of other agents or to shared goals. And a commitment that fails to perform this function will not facilitate the planning and coordination of actions involving multiple agents, nor will it increase individual agents’ motivation to contribute to other agents’ goals or to shared goals, nor will it increase their willingness to rely on other agents’ contributions.

Yet humans routinely succeed in making commitments which other humans are willing to rely on, and they routinely (though of course not always) honor their commitments. How do they do this? In some cases, humans make commitments credible by deliberately changing the material payoff structure of action options, e.g. by signing contracts that entail penalties for reneging, so that reneging is no longer an attractive outside option. In other cases, humans are motivated to honor their commitments, and/or expect others to commitments because their sense of commitment is elicited. As we noted in the previous section, the sense of commitment can be elicited and enhanced by any factor which leads one agent (ME) to expect a second agent (YOU) to make a contribution to a bringing about a goal or a desired state of affairs, or by any factor which leads that second agent (YOU) to be motivated to contribute at least in part because she believes that the first agent (ME) expects her to. Some examples may help to illustrate this.

First, ME may expect YOU to make the contribution because YOU has a reputation for making such contributions, and/or YOU may be motivated by her desire to maintain that reputation [13]. Secondly, humans may be motivated to honor commitments because doing so is associated with positive emotions (self-esteem; [14]) while reneging can cause them to experience negative emotions (cf. anticipatory guilt, [15, 16]). Moreover, people may also expect others to be motivated by such emotions, and for this reason be willing to rely on others. Thirdly, in many contexts, simple bodily cues, such as eye contact or characteristically cooperative movement dynamics, may often suffice for one agent (ME) to express her expectation that a second agent (YOU) will contribute X, and/or for the second agent (YOU) to express her intention to make that contribution [17–19].

So far, we have seen that a sense of commitment can help to motivate agents to honor commitments in the strict sense, and can lead them to expect others to honor their commitments as well, and we have also seen that a sense of commitment can also give rise to such motivations and expectations when explicit commitments are not in place. Hence, the sense of commitment is an important feature of human social life, which helps to stabilize cooperation and to facilitate coordination. For this reason, it is worth considering whether and how it may be possible to implement a sense of commitment in human–robot interaction.

## A General Challenge

One conceptual challenge arises immediately when considering how to design robots that can participate in commitments with humans. And that is that many people might simply be unprepared to believe that robots are appropriate candidates for participating in commitments. Call this the ‘But robots can’t commit!’ objection (cf. [20, p. 135]). The intuition behind such an objection may be that robots are programmed to act in specific ways and therefore not free to value action options simply because they have made a commitment to do so. Commitments involve a resistance to the pull of tempting outside options. But how could a robot ever resist the action option that it is programmed to value most highly? On the other hand, if the robot is programmed simply to do what it has committed to doing, then one might doubt whether it is doing so because of the commitment rather than simply because it is programmed to favor action options to which it has committed.

Clearly, this objection taps very fundamental and complex issues (Is free will a prerequisite for making commitments? Do *humans* even have free will? Etc.). Luckily, we believe it is both possible and productive to get around this objection by taking a pragmatic approach.

Our starting point is the hypothesis that even if a human agent does not explicitly believe that a robot can make commitments, or that she herself owes it to a robot to honor a commitment that she has made to the robot, she may nevertheless implicitly sense the opposite. This hypothesis can be motivated by considering evidence that people frequently confide in robots and experience their relationship with robots as being characterized by trust even if they do not explicitly believe that the robot is conscious or has emotions, etc. [21, 22]. Moreover, the hypothesis can also be further motivated by noting that people often act as though committed and trust other agents to do so even when they would not explicitly judge that a commitment really is in place. An example will help to illustrate this: Sam is cleaning up the living room and picks up a ball that had been lying on the floor. As it happens, his dog Woofer notices this and bounds over to him, apparently ready to play fetch. Sam was not intending to play fetch and does not particularly desire to, but may now feel obligated to, because he has generated an expectation on the part of Woofer that they will now play fetch together. In cases like this one, one may sense that a commitment is in place and act accordingly even if one would explicitly deny that there is any commitment. By the same token, one sometimes treats artifacts such as laptops and mobile phones as though they were committed to contributing to one’s goals. For example, if Frank is counting on his mobile phone to provide him with the address of the party to which he is invited, but the mobile phone fails to do so (e.g. because the battery runs out or the file has been deleted), he may become angry at the mobile phone as though it had betrayed him.

Thus, the challenge to roboticists need not be (although it could be) to design robots that *really do* engage in commitments in the strict sense or which people *explicitly believe* can engage in commitments in the strict sense, but to design robots with whom human agents tend to interact as though such commitments were in play regardless of whether human agents explicitly judge that they are in play. In other words, the challenge that we are focusing on is the challenge of implementing a *sense of commitment* in human–robot interaction.

In addressing this challenge, it will be helpful to bear two separate distinctions in mind. First of all, it is important to distinguish between the perspective of a human interactant and the perspective of the robot. Secondly, it is important distinguish the direction of the commitment—i.e. a robot being or seeming to be committed toward a human versus a human being or seeming to be committed a robot. The combination of these two distinctions yields four distinct but mutually compatible forms which a sense of commitment could take in human–robot interaction:

(i) A human has a sense of being committed toward a robot—i.e., a human agent is motivated to perform X, which is a crucial contribution to a robot’s goal or desired state (G), at least in part because she believes that the robot agent expects her to. If this is possible, it could provide new possibilities for motivating people to perform important actions such as taking their medication, practicing proper hygiene, practicing new skills that they are learning, and putting in the requisite level of effort at manufacturing jobs.
(ii) A human has a sense that a robot is committed toward her—i.e., a human agent expects a robot agent to perform X because X is a crucial contribution to the human agent’s goal or desired state (G). If this is possible, it could increase human agents’ willingness to rely upon robots and thus to perform actions which entail a risk, e.g. in rescue operations, operating theaters or military contexts.
(iii) A robot has a sense of being committed toward a human—i.e., a robot agent is motivated to perform X, which is a crucial contribution to a human agent’s goal or desired state (G), at least in part because it registers that the human agent expects it to. If this is possible, it could provide new possibilities for making robots’ motivational systems sensitive to human expectations and thereby facilitate coordination in many contexts. Moreover, it could enhance (ii) by providing a human agent with evidence to support (ii).
(iv) A robot has a sense that a human is committed toward it—i.e. a robot agent expects a human agent to perform X because X is a crucial contribution to the robot agent’s goal or desired state (G). This could enhance (i) by enabling the robot to actively elicit the human’s sense of commitment.

In order to illustrate how these forms of a sense of commitment may be implemented in human–robot interaction, the next section will be devoted to identifying and briefly discussing several concrete factors which may give rise to and/or enhance the sense of commitment in human–robot interaction.

## Implementing the Sense of Commitment in Human–Robot Interaction: Concrete Factors

The set of factors that we will be discussing is by no means exhaustive. As we have already noted, the minimal approach which we are building upon allows for a broad range of factors to qualify as modulating the degree of the sense of commitment (cf. [6]). In general, any factor which raises one agent’s (i.e. ME’s) expectation that a second agent (i.e. YOU) will perform X at least in part because X is a crucial contribution to ME’s goal or desired state (G), raises ME’s sense that YOU is committed to doing X. And any factor which raises YOU’s motivation to do X at least in part because YOU believes that ME expects this, raises YOU’s sense of being committed to ME. The aim of the current section is to illustrate how this framework can be implemented by identifying six concrete factors which appear prima facie to be feasible and potentially effective. We will discuss each of these in turn.

### Reputation Management

One factor which could play an important role in implementing a robot’s sense of being committed toward a human (i.e. form (iii) in our taxonomy) is a mechanism for reputation management. Thus, a robot could in principle be designed in such a way as to cultivate and sustain a reputation for making crucial contributions in joint actions with humans. Given that even some species of fish keep track of and actively manage reputations [23], it may not be at all far-fetched to believe that robots could also exhibit an analogous capacity.

By interacting with such a robot over time, a human could build up an expectation that the robot would make crucial contributions to the human’s goals and desired states, and consequently be willing to rely on the robot. In other words, it may be possible to implement form (ii) from our taxonomy, i.e. to enhance a human’s sense that the robot is committed to her. This may also be achieved by providing humans with appropriate background information about the robot, e.g. third-parties could inform people that they had found the robot to have a pronounced sense of commitment toward people and/or to expect the same of its human collaborators (on the top-down effects of humans’ background beliefs about robots with whom they are interacting, see [24]). In this connection, it is worth noting that previous research has shown that such gossip can profoundly influence people’s willingness to cooperate in various contexts [25]. Indeed, in the context of a trust game, [26] observed the same phenomenon even when participants could have accessed the relevant information by direct observation. And [27] found that participants made more risky investment decisions with partners when they had been informed by a third-party that the partner had a good reputation.

### Emotions

Emotions are of central importance for the sense of commitment for at least two distinct reasons. First, emotions may be crucial in motivating agents to honor commitments. In particular, [28] has argued that the possibility of reneging on a commitment and taking some tempting alternative option may appear less tempting to people insofar as they anticipate the likely emotional outcomes of reneging, such as guilt, shame, anger, disgust, etc. [15, 16]). Thus, such emotions—or functional equivalents thereof—in robots may provide a motivation to do X, which may help in implementing a robot’s sense of being committed to a human, as well as a human’s sense that the robot is committed to her. Secondly, one agent’s emotional expressions can signal a sense of commitment to a second agent [29]. For example, if YOU expresses frustration when ME’s attempts to bring about G are unsuccessful, this could enhance ME’s sense that YOU is committed. Both of these functions of emotions could in principle be implemented in robots.

With respect to the motivational role of emotions, the challenge would be to implement functional states in robots which play this role. In other words, the challenge need not be to implement real emotions. Instead, robots could be designed with states that play the functional roles of guilt, fear or disgust in motivating it to honor (implicit and explicit) commitments. If humans are informed of this and/or have the opportunity to observe it, it may increase their willingness to rely on the robot. It is interesting in this context to note that humans do tend to ascribe feelings to robots. For example, [30] reported that human participants reacted empathically (i.e. rejoicing or commiserating) to images of a dinosaur-robot either being treated in a friendly way or being tortured.

With respect to the implementation of emotional expressions as signals of a sense of commitment, one challenge is that robots have different bodies from humans and may therefore be unable to express emotions in a way that elicits the same type of response from human interactants as human expressions of emotions. Indeed, in view of the well-known uncanny valley effect [31], it may be counterproductive to design robots such that they express emotions in a way that too closely parallels human emotional expressiveness. In order to address this challenge, it would be useful to identify particular forms of expression that work well in humans. For example, perhaps facial expressions of emotion would not be the most feasible (in view of the uncanny valley effect), but bodily posture or tone of voice might be more effective. By the same token, it could be useful to develop robots’ capacity to monitor and respond sensitively to *humans’* emotional expressions. As [32] have shown, people tend to have more favorable impressions of robots that are responsive to their (i.e. humans’) emotionally meaningful disclosures.

### Eye Contact

There are several interesting studies showing the importance of eye contact in human–human joint actions. For example, [33] devised a stag hunt game in which four-year-old children could choose to attempt to attain a small prize (i.e. the hare, in this case represented by marginally cool stickers) or a large prize (i.e. the stag, in this case represented by much cooler stickers). In order to attain the large prize, they had to operate a large lever which could only be operated if the other player also opted to go for the larger prize, whereas they could obtain the smaller prize by operating a smaller lever by themselves. The interesting finding was that the children were far more likely to go for the large prize if there had been eye contact with the other player, i.e. signaling an implicit commitment to coordinate efforts in order to obtain the larger prize. The notion that eye contact generates an implicit commitment to be cooperative gains further support from and interesting study by [34]. In a gaze-cueing paradigm, they presented participants with faces gazing either to the right or to the left, and participants that either reliably cued, or reliably failed to cue, the location of an object which appeared shortly thereafter, and which participants had the task of detecting. What they found was that participants were equally likely to follow the gaze of the seen faces, regardless of how helpful this was, but that they tended to rate the unhelpful faces as less trustworthy than the helpful faces—as though the unhelpful faces had violated an implicit commitment.

These findings reveal that eye contact can under certain circumstances increase one agent’s (ME’s) expectation that a second agent (YOU) will perform X, which is a crucial contribution to G, a goal or desired state of ME. In addition, they indicate that eye contact can also increase YOU’s motivation to perform X by leading her to believe that ME expects this.

An obvious challenge that may arise in using eye contact this way in human–robot interaction is that robots’ specific bodily forms may make it difficult for them to give such signals as easily as humans (but see [35]). However, it is worth noting that human participants respond to cartoon faces [36, 37] much in the same way as they respond to human faces in gaze cueing paradigms, i.e. they tend to follow gaze direction. Indeed, [38] found that even young children tend to follow the gaze direction of robots as long as those robots act in a way that is contingent upon their actions, and thus appear to be contingently responsive to them. Intriguingly, in one experiment, the robots did not even have faces but merely front and rear sides. Hence, it could be possible to design robots that could engage in eye contact even without having faces, and certainly without having human-like faces.

### Signaling

There has also been a great deal of research in recent years on what has been called ’signaling’, i.e. a form of characteristically cooperative movements dynamics in joint action [12, 19, 39, 40]. For example, [19] found that participants tended to sacrifice efficiency of movement in order to make their movements more easily and quickly predictable for their partners. Insofar as this type of signaling constitutes an investment in the joint action, and demonstrates a willingness to coordinate with one’s partner, and also an expectation that the one’s partner will remain engaged. As a result, it could enhance the partner’s YOU’s sense of being committed until the goal is reached. If so, it is plausible that signaling could also have such effects in human–robot interactions. After all, there is no need to have the same bodily shape as a human in order to adapt one’s movements to make them more predictable, as [19] results indicate. Indeed, work by [17] has shown that it is possible to identify bodily cues that correlate with trust in dyadic interactions, and to design robots to exhibit and to identify such cues.

### Coordination

While the foregoing remarks about emotional expression and signaling pertain to bodily cues exhibited by individuals within interactions, there is also research suggesting that the *inter*personal dynamics within social interactions may also modulate the sense of commitment Specifically, Michael et al. (in preparation) contrasted a condition with a high degree of coordination (e.g. two agents form a chain to clean up a pile of sand, with one agent scooping up a bucket of sand and passing it to the second agent, who then empties it into a container) with a separate condition involving a low degree of coordination (the same two agents work alongside each other without forming a chain), and found evidence that the former condition generated a greater sense of commitment. According to the minimal framework sketched above, coordination generates commitment because, when one agent (ME) performs a subtask depending upon the second agent (YOU) performing her subtask, she expresses her expectation that the second agent will in fact perform her subtask, and thereby elicits YOU’s sense of commitment.

### Mutual Supportiveness

As philosophers have observed [2, 41–44], characteristically human ‘shared cooperative activity (SCA)’ often requires participants to carry out complementary, or meshing, subplans, and to be able and willing to flexibly adjust their subplans in order to bring about their shared goals. For example, if two individuals want to paint a house together, they will have to agree not only on the shared goal of painting the house but on the specific subplans which they will implement in order to achieve their shared goal; one of them may buy the paint while the other one buys the brushes, etc. On Bratman’s [41] influential analysis, the shared intention to achieve a shared goal is formed in accordance with and because of such meshing subplans. In addition to a shared goals and meshing subplans, though, [41] also specifies a further criterion, namely mutual supportiveness^3^.

How might this be implemented in the case of human–robot interaction? To begin with, let us note that there seems to be no particular reason why robots should have specific difficulties with identifying how goals can be achieved by means of distinct meshing subplans, as [47] have already demonstrated (cf. also [48]). After all, it seems plausible to assume that a robot can coordinate with other (human or non-human) agents, provided that the task is well-defined. Some additional challenge could be presented in designing robots that can flexibly adapt when a human partner appears to need additional support, or to expect a human to flexibly offer additional support. But if this is possible, the latter may indicate a high level of motivation [i.e. form (iii) of the sense of commitment], whereas the former could indicate an expectation on the robot’s part that the human will make crucial contributions, thus enhancing the robot’s sense that the human is committed [form (iv)] and potentially also the human’s sense of being committed [form (i)].

The feasibility of both of these capacities (i.e. to offer and to expect mutual support) is illustrated by a recent project by Clodic et al. [49]. Clodic and colleagues designed an interactive robot guide, ‘Rackham’, who made explicit agreements with museum visitors to guide them through an exhibition, and who was able to monitor whether his ‘clients’ were following him, to wait for them, to adjust his pace to theirs, etc.

## Conclusions

In this paper we have discussed some of the central challenges that roboticists might face in implementing a sense of commitment in human–robot interaction. Obviously, there are deeper philosophical questions which our pragmatic approach has not addressed. Specifically, we have said nothing about whether a human agent would be right in thinking that a robot interactant had the capacity to honor commitments and to demand the same in return. In other words, there is a further question about whether and under what conditions robots can *really* engage in commitments. To be sure, answering this question would require a larger investigation of deep conceptual issues. Whatever one’s assessment of this question, though, it seems clear that the potential is immense for robots that exhibit and/or elicit a sense of commitment—i.e. such that (i) humans agents are motivated by a sense of commitment toward them, (ii), human agents expect them to be motivated by a sense of commitment toward human agents, (iii) they are motivated by a sense of commitment toward human agents, and/or (iv) they expect human agents to be motivated by a sense of commitment toward them.

## Footnotes

1 This standard conception helps to make clear how commitment differs from the related phenomenon of trust: unlike trust, commitments are linked to *specific *actions. Jennifer may trust Susie *in general* to behave in a responsible manner, to honor her commitments, etc. But her trust in Susie does not yet suffice for her to expect Susie to to pick up the kids today or for her to judge that Susie is obligated to do so. It is not until Susie has agreed to perform this particular action that a commitment in the strict sense arises to perform this particular action. Thus, while the concept of trust picks out a general disposition to expect an agent to behave in a manner that supports one’s interests and well-being, the concept of commitment picks out specific obligations to perform specific actions, which arise because of agreements that have intentionally been made.

2 In saying that the contribution is crucial, we mean that it is a necessary component of a particular strategy for bringing about G.

3 The motivation to offer mutual support to each other when needed may underpin a ‘mutual assumption of helpfulness,’ which is central to the notion of shared intentionality ([45, 46], cf. also Salice and Satne in preparation).
